# Combination Therapy of Human Umbilical Cord Blood Cells and Granulocyte Colony Stimulating Factor Reduces Histopathological and Motor Impairments in an Experimental Model of Chronic Traumatic Brain Injury

**DOI:** 10.1371/journal.pone.0090953

**Published:** 2014-03-12

**Authors:** Sandra A. Acosta, Naoki Tajiri, Kazutaka Shinozuka, Hiroto Ishikawa, Paul R. Sanberg, Juan Sanchez-Ramos, Shijie Song, Yuji Kaneko, Cesar V. Borlongan

**Affiliations:** 1 Center of Excellence for Aging and Brain Repair, Department of Neurosurgery and Brain Repair, University of South Florida College of Medicine, Tampa, Florida, United States of America; 2 Office of Research and Innovation, University of South Florida, Tampa, Florida, United States of America; 3 James Haley Veterans Affairs Medical Center, Tampa, Florida, United States of America; 4 Department of Neurology, University of South Florida, Tampa, Florida, United States of America; 5 Department of Molecular Pharmacology and Physiology, University of South Florida, Tampa, Florida, United States of America; French Blood Institute, France

## Abstract

Traumatic brain injury (TBI) is associated with neuro-inflammation, debilitating sensory-motor deficits, and learning and memory impairments. Cell-based therapies are currently being investigated in treating neurotrauma due to their ability to secrete neurotrophic factors and anti-inflammatory cytokines that can regulate the hostile milieu associated with chronic neuroinflammation found in TBI. In tandem, the stimulation and mobilization of endogenous stem/progenitor cells from the bone marrow through granulocyte colony stimulating factor (G-CSF) poses as an attractive therapeutic intervention for chronic TBI. Here, we tested the potential of a combined therapy of human umbilical cord blood cells (hUCB) and G-CSF at the acute stage of TBI to counteract the progressive secondary effects of chronic TBI using the controlled cortical impact model. Four different groups of adult Sprague Dawley rats were treated with saline alone, G-CSF+saline, hUCB+saline or hUCB+G-CSF, 7-days post CCI moderate TBI. Eight weeks after TBI, brains were harvested to analyze hippocampal cell loss, neuroinflammatory response, and neurogenesis by using immunohistochemical techniques. Results revealed that the rats exposed to TBI treated with saline exhibited widespread neuroinflammation, impaired endogenous neurogenesis in DG and SVZ, and severe hippocampal cell loss. hUCB monotherapy suppressed neuroinflammation, nearly normalized the neurogenesis, and reduced hippocampal cell loss compared to saline alone. G-CSF monotherapy produced partial and short-lived benefits characterized by low levels of neuroinflammation in striatum, DG, SVZ, and corpus callosum and fornix, a modest neurogenesis, and a moderate reduction of hippocampal cells loss. On the other hand, combined therapy of hUCB+G-CSF displayed synergistic effects that robustly dampened neuroinflammation, while enhancing endogenous neurogenesis and reducing hippocampal cell loss. Vigorous and long-lasting recovery of motor function accompanied the combined therapy, which was either moderately or short-lived in the monotherapy conditions. These results suggest that combined treatment rather than monotherapy appears optimal for abrogating histophalogical and motor impairments in chronic TBI.

## Introduction

Traumatic brain injury (TBI) produces debilitating conditions that affect millions worldwide [1]. Recent data show that in the United States alone more than 1.7 millions, including military personnel, sustain a TBI every year [1], [2]. Regardless of the severity of the trauma, motor, behavioral, intellectual and cognitive disabilities will be manifested as both short- and long-term [3], [4], [5], [6], [7], [8]. In moderate to severe trauma, TBI survivors present with chronic disabilities associated with loss of primary cerebral parenchymal tissues, secondary cell death including apoptosis, and exacerbated neuroinflammation [9], [10], [11]. Among the worst outcomes, sensorimotor dysfunctions, massive hippocampal cell death, learning and memory impairments, aphasia, anxiety, and dementia are the most prevalent [12], [13], [14], [15], [16]. At present, clinical treatments are limited and the few that have been utilized have proven to be ineffective in most of the TBI cases [17], [18], [19]. Preclinical studies have demonstrated that adult stem/progenitor cells transplantation is a promising therapeutic intervention for TBI [20], [21]. Bone marrow stromal cells (BMSC), adipose derived stem cells (ADSC), amniotic fluid stem cells (AFSC) and the mononuclear fraction of human umbilical cord blood (hUCB) have shown neuroprotective properties by decreasing inflammation, brain tissue loss, promoting neurogenesis, and rescuing neurological functions such as learning and memory in experimental models of chronic TBI [20], [21], [22], [23], [24]. However, the injured micro-environment limits their regenerative potential [19], [25]. For instance, TBI victims suffer from brain oxygen depletion, vasogenic edema, and secondary injury signals including reactive oxygen species, exacerbated activated MHCII+ cells, astrogliosis and pro-inflammatory cytokines such as, but not limited to, IL-1beta, TNF-alpha which can accumulate in the area of injury leading to decreased survival of transplanted adult stem cells [11], [25], [26]. The use of combined therapies stands as a promising technique to overcome molecular aberrations while enhancing the adult stem cells' therapeutic potential in chronic TBI [27], [28].

Colony stimulating factors (CSF), also called haemopoietic growth factors, regulate the mobilization, proliferation, and differentiation of bone marrow cells. Growth factors such as granulocyte colony stimulating factor (G-CSF), granulocytes-macrophages colony stimulating factor (GM-CSF), colony stimulating factor-1(CSF-1), and erythropoietin are currently being investigated as therapeutics for cancer, certain autoimmune diseases, ischemic insults and neurodegenerative diseases [10], [29], [30]. Recent evidence suggests that G-CSF affords beneficial effects against central nervous system (CNS) conditions such as stroke and Alzheimer's disease [30], [31], [32]. Short-term treatment of systemic G-CSF significantly improved cognition accompanied by reduced central and peripheral inflammation, enhanced neurogenesis and decreased the amyloid deposition in the hippocampus and entorhinal cortex in both mice and rats experimental models of Alzheimer's disease [30], [32]. Similarly, G-CSF, together with stem cell factor, restored neural circuits by facilitating anatomical connections of dendritic spines and branches with the adjacent infracted area of experimental stroke [31]. Recent clinical trials of G-CSF treatment in stroke patients have been proven safe [33], but efficacy remains inconclusive [10].

In the present *in vivo* study, we assessed if hUCBC transplantation combined with G-CSF treatment afforded enhanced neuroprotection in a rat CCI model of moderate TBI in the long term using validated TBI immunohistochemical parameters of hippocampal cell loss, neuroinflammatory response, and neurogenesis. Here we report synergistic effects of hUCB transplantation and G-CSF treatment as evidenced by highly effective sequestration of hippocampal cell loss, much more robust reduction in neuroinflammation and massive neurogenesis in the TBI brain compared to stand alone therapies.

## Methods

### Subjects

Experimental procedures were approved by the University of South Florida Institutional Animal Care and Use Committee (IACUC). All male rats were housed under normal conditions (20°C, 50% relative humidity, and a 12-h light/dark cycle). Normal light/dark cycle was employed. A separate cohort of animals, saline group, (n = 7); G-CSF group, (n = 8); hUCB group (n = 8); hUCB+G-CSF (n = 8) underwent the same experimental above and subjected to behavioral tests to determine the functional effects of hUCB and G-CSF. All behavioral testing was done during the light cycle at the same time across testing days. Necessary precautions were taken to reduce pain and suffering of animals throughout the study. All studies were performed by personnel blinded to the treatment condition.

### Surgical procedures

Ten-week old Sprague–Dawley rats (n = 55) were subjected to TBI using a controlled cortical impactor (CCI; Pittsburgh Precision Instruments, Inc, Pittsburgh, PA). Deep anesthesia was achieved using 1–2% isoflurane in nitrous oxide/oxygen (69/30%) using a nose mask. All animals were fixed in a stereotaxic frame (David Kopf Instruments, Tujunga, CA, USA). TBI injury surgeries consisted of animals subjected to scalp incision to expose the skull, and craniectomy. An electric drill was used to perform the craniectomy of about 2.5 mm radius with coordinates calculated from +0.2 anterior and −0.2 mm lateral right from bregma [34]. After craniotomy the brain was impacted at the fronto-parietal cortex with a velocity of 6.0 m/s reaching a depth of 1.0 mm below the dura matter layer and remained in the brain for 150 milliseconds (ms). The impactor rod was angled 15° degrees vertically to maintain a perpendicular position in reference to the tangential plane of the brain curvature at the impact surface. A linear variable displacement transducer (Macrosensors, Pennsauken, NJ), which was connected to the impactor, measured the velocity and duration to verify consistency. The analgesic ketoprofen (5 mg kg–1) was administered postoperatively. Rats were kept under close supervision.

### Intravenous administration of G-CSF and hUCB cells

One week post-TBI CCI surgery, rats were anesthetized with 1–2% isoflurane in nitrous oxide/oxygen (69/30%) using a nose mask. Four different groups, (n = 6), of TBI rats were treated intravenously through the jugular vein with either saline alone (500 µl of sterile saline), G-CSF (300 µg/kg in 500 µl of sterile saline), hUCB+saline (4×10^6^ viable cells supplied by Saneron CCEL Therapeutics, Inc., in 500 µl of sterile saline), or hUCB+G-CSF (4×10^6^ viable cells in 500 µl of sterile saline and 300 µg/kg G-CSF in 500 µl of sterile saline) at 7 days post TBI.

### Histology

Under deep anesthesia, rats were sacrificed 8 weeks after TBI surgery. For transcardial perfusion, rats were placed on their backs, and blunt forceps were used to cut through the body cavity. The opening was extended laterally until reaching the rib cage. Rib cage was cut, and sternum was lifted to expose the heart. The heart was held gently, and the needle was inserted ¼ inch into the left ventricle. The right atrium was cut using the irridectomy scissors. Through the ascending aorta, approximately 200 ml of ice cold phosphate buffer saline (PBS) followed by 200 ml of 4% paraformaldehyde (PFA) in PBS were used for brain perfusion. Brains were removed and post-fixed in the same fixative at 4°C for 24 hours followed by 30% sucrose in phosphate buffer (PB) for 1 week. Brains were frozen at −24°C, mounted onto a 40 mm specimen disk using embedding medium. Coronal sectioning was carried out at a thickness of 40 µm by cryostat. H& E and Immunostainings were done on every 6^th^ (1/6) coronal section spanning the frontal cortex and the entire dorsal hippocampus.

### Hematoxylin and eosin analysis

Hematoxylin and eosin (H&E) staining was performed to confirm the core impact injury of our TBI model. As shown in our previous studies [11], [35], [36], [37], we also demonstrated here that the primary damage produced by the CCI TBI model was to the fronto-parietal cortex. In addition, H&E staining was analyzed in the hippocampus to reveal surviving neurons. . In all animals, sections were anatomically matched. Series of 6 sections per rat were processed for staining. H&E staining was done on every sixth coronal section spanning the dorsal hippocampus, starting at coordinates AP-2.0 mm and ending at AP-3.8 mm from bregma. Cells presenting with nuclear and cytoplasmic staining (H&E) were manually counted in the CA3 neurons. CA3 cell counting spanned the whole CA3 area, starting from the end of hilar neurons to the beginning of curvature of the CA2 region in both the ipsilateral and contralateral side. In order to calculate the % of surviving neurons on the CA3, the % of CA3+ neurons on the ipsilateral side are compared to the contralateral side to TBI hemispheres. Sections were examined with Nikon Eclipse 600 microscope at 20× All data are represented as mean values ±SEM, with statistical significance set at p<0.05.

### Immunohistochemistry

Staining for DCX and OX6 was conducted on separate sets of section of every 6^th^ coronal section throughout the entire striatum and dorsal hippocampus. In all animals, sections were anatomically matched. For the DCX staining normal horse serum was used. For the MHCII (OX6) staining normal goat serum was used. Sixteen free-floating coronal sections (40 µm) were washed 3 times in 0.1M phosphate-buffered saline (PBS) to clean the section from cryoprotectant. Thereafter, all section were incubated in 0.3% hydrogen peroxide (H_2_O_2_) solution for 20 minutes and washed 3 times with PBS for 10 minutes each wash. Next, all sections were incubated in blocking solution for 1 hour using PBS supplemented with 3% normal serum and 0.2% Triton X-100. Sections were then incubated overnight at 4°C with either goat anti rat DCX (1∶150 immature neuronal marker doublecortin or DCX; Santa Cruz; cat#sc8066), or mouse anti rat MHCII (OX6) (major histocompatibility complex or MHC class II; 1∶750 BD; cat# 554926) antibody markers in PBS supplemented with 3% normal serum and 0.1% triton X-100. Sections were then washed 3 times with PBS and incubated in biotinylated horse anti-goat secondary antibody (1∶200; Vector Laboratories, Burlingame, CA)for the DCX staining, or goat anti-mouse goat secondary antibody (1∶200; Vector Laboratories, Burlingame, CA) in PBS supplemented with normal serum, and 0.1% Triton X-100 for 1 hour. Next, the sections were incubated for 60 minutes in avidin–biotin substrate (ABC kit, Vector Laboratories, Burlingame, CA) and washed 3 times with PBS for 10 minute each wash. All sections were then incubated for 1 minute in 3,30-diaminobenzidine (DAB) solution (Vector Laboratories) and wash 3 times with PBS for 10 minutes each wash. Sections were then mounted onto glass slides, dehydrated in ascending ethanol concentration (70%, 95%, and 100%) for 2 minutes each and 2 minutes in xylenes, then cover-slipped using mounting medium.

### Stereological analysis

Unbiased stereology was performed on brain sections immunostained with OX6, and DCX. Sets of 1/6 section, ∼16 systematically random sections, of about 240 µm apart, were taken from the brain spanning AP – 0.2 mm to AP – 3.8 mm in all 24 rats. Activated MHCII+ cells, and differentiation into immature neurons were visualized by staining with OX6, DCX, respectively. Positive stains were analyzed with a Nikon Eclipse 600 microscope and quantified using Stereo Investigator software, version 10 (MicroBrightField, Colchester, VT). The estimated volume of OX6-positive cells was examined using the Cavalieri estimator probe of the unbiased stereological cell technique revealing the volume of OX6 in the cortex, striatum, thalamus, fornix, cerebral peduncle, and corpus callosum. The literature supports the concept of the Cavalieri principle as a stereological technique used to estimate the volume of structures and regions with arbitrary shape and size, and not only to spherical structures or regions [35], [36], [37]. DCX positive cells were counted within the subgranular zone (SGZ) of the dentate gyrus of the hippocampus, and within the subventricular zone (SVZ) of the lateral ventricle, in both hemispheres (ipsilateral and contralateral), using the optical fractionator probe of unbiased stereological cell counting technique. The sampling was optimized to count at least 300 cells per animal with error coefficients less than 0.07. Each counting frame (100×100 µm for OX6, and DCX) was placed at an intersection of the lines forming a virtual grid (175×175 µm), which was randomly generated and placed by the software within the outlined structure. Section thickness was measured in all counting sites. For the DCX+ cells analysis in the DG and SVZ, the % of DCX+ neurons on the ipsilateral side are compared to the contralateral side to TBI hemispheres.

### Behavioral Tests

Elevated body swing test (EBST) involved handling the animal by its tail and recording the direction of the swings [38]. The test apparatus consisted of a clear Plexiglas box (40×40×35.5 cm). The animal was gently picked up at the base of the tail, and elevated by the tail until the animal's nose is at a height of 2 inches (5 cm) above the surface. The direction of the swing, either left or right, was counted once the animals head moves sideways approximately 10 degrees from the midline position of the body. After a single swing, the animal was placed back in the Plexiglas box and allowed to move freely for 30 seconds prior to retesting. These steps were repeated 20 times for each animal. Normally, intact rats display a 50% swing bias, that is, the same number of swings to the left and to the right. A 75% swing bias towards one direction was used as criterion of TBI motor deficit. For assessment of motor balance and coordination, the animals were tested in the rotorod test following the procedures described elsewhere [39]. The rotorod treadmill (Accuscan, Inc., Columbus, OH, USA) produced motor balance and coordination data which were generated by averaging the scores (total time spent on treadmill divided by 5 trials) for each animal during training and testing days. Each animal was placed in a neutral position on a cylinder (3 cm and 1 cm diameter for rats and mice, respectively) then the rod was rotated with the speed accelerated linearly from 0 rpm to 24 rpm within 60 s, and the time spent on the rotrod was recorded automatically. The maximum score given to an animal was fixed to 60. For training, animals were given 5 trials each day and declared having reached the criterion when they scored 60 in 3 consecutive trials. For testing, animals were given 3 trials and the average score on these 3 trials was used as the individual rotorod score.

### Statistical analysis

For immunohistochemical data analyses, contralateral and ipsilateral corresponding brain areas were used as raw data providing 4 sets of data per treatment condition (TBI+saline alone, G-CSF+saline, hUCB+G-CSF, or hUCB+saline), therefore two-way and one-way analysis of variance (ANOVA) was used for group comparisons, followed by subsequent pairwise comparisons using post hoc Bonferonni test, which included analyses of any differences between treatments, as well as between contralateral and ipsilateral hemispheres. For behavioral data analyses, repeated measures of ANOVA and post hoc Bonferroni's *t*-tests for each time point were used to evaluate statistical differences between treatment groups. For all analyses, differences were considered significant at *p*<0.05. All values are expressed as mean±SEM.

## Results

### Treatment with hUCB and G-CSF monotherapy, and hUCB+G-CSF combined therapy ameliorate TBI–induced neuroinflammation

**Figure 1:**
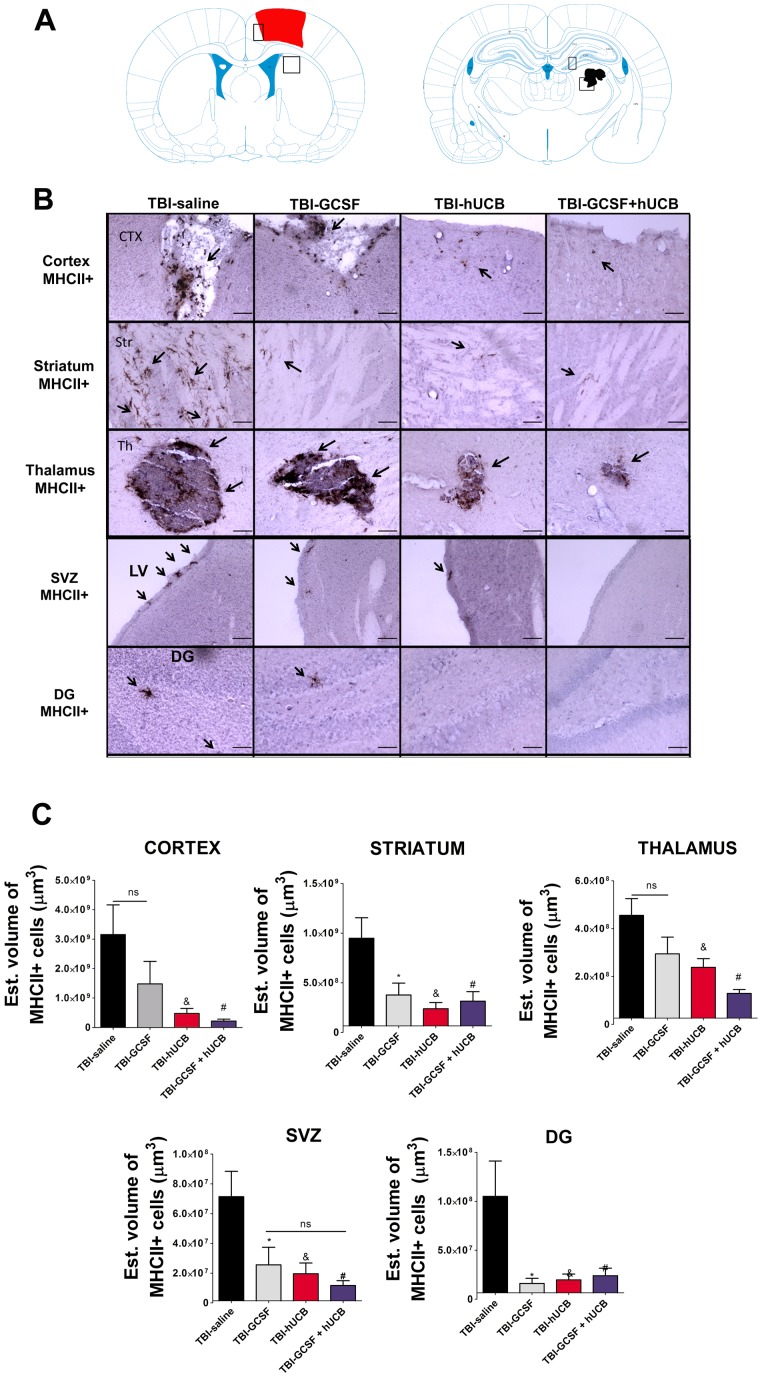
Monotherapy of hUCB or G-CSF, and hUCB+G-CSF combined therapy ameliorate TBI–induced neuroinflammation in gray matter areas. Downregulation of activated microglial cells in the ipsilateral side of cortical and subcortical gray matter regions after treatment with hUCB alone, G-CSF alone, and combined hUCB+G-CSF relative to saline. Diagram of a coronal section shows the lesion area in red. The squares indicate the region of interest for analyses (Fig. 1A). Photomicrographs of gray matter areas of cortex, striatum, thalamus, SVZ and DG of coronal sections from all four groups (Fig. 1B). Arrows indicate positive staining for activated microglia cells. Quantification of OX-6 immunostaining reflects estimated volume of activated microglia cells of cortex, striatum, thalamus, SVZ, and DG (Fig. 1C). Cortex F3, 20 = 4.913, p<0.0001; striatum F3,20 = 6.466, p<0.0001; thalamus F3,20 = 8.785, p<0.0001; SVZ F3,20 = 6.543, p<0.0001; DG F3,20 = 4.587, p<0.0001. Scale bar in B = 1 µm. * = significant difference between TBI-saline and TBI-G-CSF; & = significant difference between TBI-saline and TBI-hUCB; # = significant difference between TBI-saline and TBI-hADSC; ns = no significance. Significance at p's<0.05.

**Figure 2:**
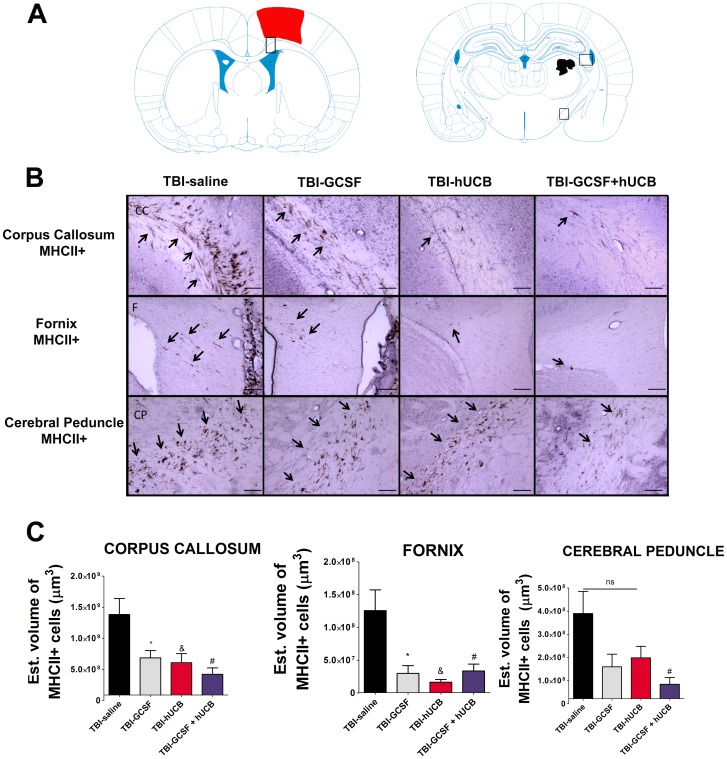
Monotherapy of hUCB or G-CSF, and hUCB+G-CSF combined therapy ameliorate TBI–induced neuroinflammation in white matter areas. Downregulation of activated microglia cells in the ipsilateral side of white matter axonal regions after treatment with hUCB alone, G-CSF alone, and combined hUCB+G-CSF relative to TBI-saline. Diagram of a coronal section shows the axonal lesion areas. The squares indicate the region of interest for analyses (Fig. 2A). Photomicrographs of white matter areas: corpus callosum, fornix, and cerebral peduncle of coronal sections from all four groups (Fig. 2B). Arrows indicate positive staining for activated microglia cells. Quantification of OX-6 immunostaining reflects estimated volume of activated microglia cells in corpus callosum, fornix, and cerebral peduncle (Fig. 2C). Corpus callosum, F3,20 = 14.6, p<0.0001; fornix, F3,20 = 9.017, p<0.0001; cerebral peduncle, F3,20 = 4.638, p<0.0001. Scale bar for B = 1 µm. * = significant difference between TBI-saline and TBI-G-CSF; & = significant difference between TBI-saline and TBI-hUCB; # = significant difference between TBI-saline vs TBI-hADSC; ns = no significance. Significance at p's<0.05.

The brain regions examined for TBI-induced neuroinflammation included the gray matter areas of cortex, striatum, thalamus, SVZ, and DG of the hippocampus, while the white matter areas analyzed were corpus callosum, fornix, and cerebral peduncle (Fig. 1A, 2A]. ANOVA revealed overall significant treatment effects on inflammation as evidenced by OX-6 immunostaining in all brain regions examined here as follows: cortex F_3, 20_ = 4.913, p<0.0001; striatum F_3,20_ = 6.466, p<0.0001; thalamus F_3,20_ = 8.785, p<0.0001; SVZ F_3,20_ = 6.543, p<0.0001; DG F_3,20_ = 4.587, p<0.0001; corpus callosum, F_3,20_ = 14.6, p<0.0001; fornix, F_3,20_ = 9.017, p<0.0001; cerebral peduncle, F_3,20_ = 4.638, p<0.0001. Posthoc test analysis revealed a robust upregulation of activated microglia cells when comparing the total estimated volume of MHCII+ cells in the ipsilateral hemisphere of rats exposed to chronic TBI and treated with saline alone to their contralateral side across all gray and white matter areas analyzed (p<0.0001), except in the corpus callosum area (p>0.05).

The estimated volume of MHCII+ activated cells was quantified using stereological techniques. One way ANOVA revealed a significant treatment effects in the ipsilateral cortex and subcortical gray matter areas As follows: cortex F_3, 20_ = 4.869, p<0.0075; striatum F_3,20_ = 5.107, p<0.0025; thalamus F_3,20_ = 7.044,p<0.0012; SVZ F_3,20_ = 6.543, p<0.0019; DG F_3,20_ = 5.237, p<0.0061. Post hoc Bonferroni's test revealed that monotherapy of hUCB cells and the combined therapy of hUCB+G-CSF significantly decreased the TBI-associated upregulation of MHCII+ activated cells in the cortex, striatum, thalamus, SVZ, and DG relative to rats exposed to chronic TBI treated with saline alone (p<0.05) (Fig. 1B, C). G-CSF monotherapy was also effective in reducing activated MHCII+ cells in most gray matter areas analyzed (p<0.05), except in the cortical and thalamic area compared to treatment of saline alone (p>0.05) (Fig. 1C).

Similar analyses of OX-6 neuroinflammation demonstrated significant treatment effects in several white matter areas ipsilateral to TBI injury (Fig. 2A). ANOVA revealed statistical significance in the following white matter areas as follows: corpus callosum, F_3,20_ = 6.506, p<0.0018; fornix, F_3,20_ = 8.324, p<0.0005; cerebral peduncle, F_3,20_ = 4.733, p<0.0088. Post hoc Bonferroni's test analysis revealed that combined therapy of hUCB+G-CSF decreased the TBI-associated upregulation of activated MHCII+ cells in corpus callosum, fornix, and cerebral peduncle ipsilateral to injury when compared to rats exposed to TBI treated with saline alone (p<0.05) (Fig. 2B, C). hUCB cells alone and G-CSF alone were effective at significantly suppressing activated MHCII+ cells only in two white matter areas, namely the corpus callosum and fornix relative to rats exposed to chronic TBI treated with saline alone (p<0.05) (Fig. 2C).

### hUCB and G-CSF monotherapy, and combined hUCB+G-CSF attenuate TBI-induced impairment in endogenous neurogenesis

**Figure 3:**
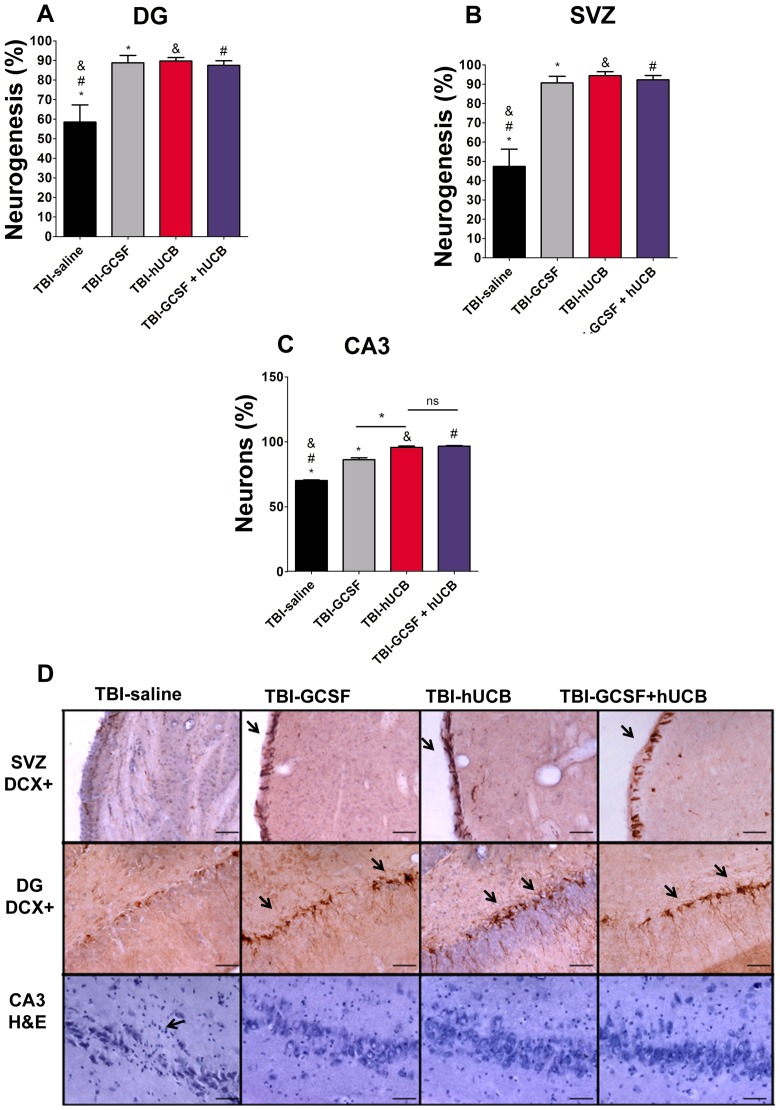
hUCB and G-CSF monotherapy, and combined hUCB+G-CSF attenuate TBI-induced impairment in endogenous neurogenesis and cell loss in rats exposed to chronic TBI. Significantly enhanced neural differentiation in the DG and SVZ and increased cell survival in CA3 after hUCB or G-CSF monotherapy, and the combined therapy of hUCB+G-CSF relative to saline alone. All 3 treatment conditions percent neurogenesis in the DG (Fig. 3A). Percent neurogenesis in the SVZ (Fig. 3B). Percentage of neuronal survival in the CA3 region of the hippocampus (Fig. 3C). Photomicrographs of SVZ, DG, and CA3 region (Fig. 3D) Top panel arrows indicate positive staining for neurogenesis in SVZ and DG respectivately. Arrow on bottom panel indicates CA3 pyramidal cell loss in TBI-saline (Fig. 3D). F_3,20_ = 159.3, p<0.0001. Scale bars for Figure D = 50 µm. * = significant difference between TBI-saline and TBI G-CSF; & = significant difference between TBI-saline and TBI-hUCB; # = significantly difference between TBI-saline and TBI-hADSC; ns = no significance. Significance at p's<0.05.

ANOVA revealed significant treatment effects on neurogenesis in the neurogenic DG (F_3, 20_ = 9.107, p<0.0005). Post hoc Bonferroni's test analysis revealed that hUCB and G-CSF monotherapy, and the combined therapy of hUCB+G-CSF significantly enhanced endogenous neurogenesis in the DG in our model of chronic TBI compared to injured rats treated with saline alone (p<0.05) (Fig. 3A). Moreover, ANOVA revealed significant treatment effects on neurogenesis in the other neurogenic site, SVZ (F_3, 20_ = 20.00, p<0.0001) (Fig. 3B). Post hoc Bonferroni's test analysis revealed a significant upregulation of neurogenesis in the SVZ of chronically TBI-exposed rats treated with either hUCB and G-CSF monotherapy, and the combined therapy of hUCB+G-CSF compared to injured rats treated with saline alone (p<0.05) (Fig. 3D)

### hUCB monotherapy and combined hUCB+G-CSF robustly, while G-CSF alone moderately decrease hippocampal cell loss in rats exposed to chronic TBI

ANOVA revealed significant treatment effects on TBI-induced hippocampal cell loss (ANOVA, F_3,20_ = 159.3, p<0.0001). Treatment with hUCB cell monotherapy and the combined therapy of hUCB+G-CSF significantly rescued the neuronal cells in the CA3 region of the hippocampus (Fig. 3C). There was a significant survival of cells in CA3 regions in rats exposed to chronic TBI and treated with either hUCB cells alone or combined hUCB+G-CSF compared with injured rats treated with G-CSF alone or saline (p's<0.05). Although not as robust an effect as hUCB alone and combined hUCB+G-GSCF, quantitative analyses also revealed a significant increase in survival of CA3 neurons in injured rats treated with G-CSF monotherapy relative to those treated with saline (p<0.05) (Fig. 3C).

### Treatment with hUCB and G-CSF monotherapy, and hUCB+G-CSF combined therapy promote behavioral recovery in chronic TBI animals

**Figure 4:**
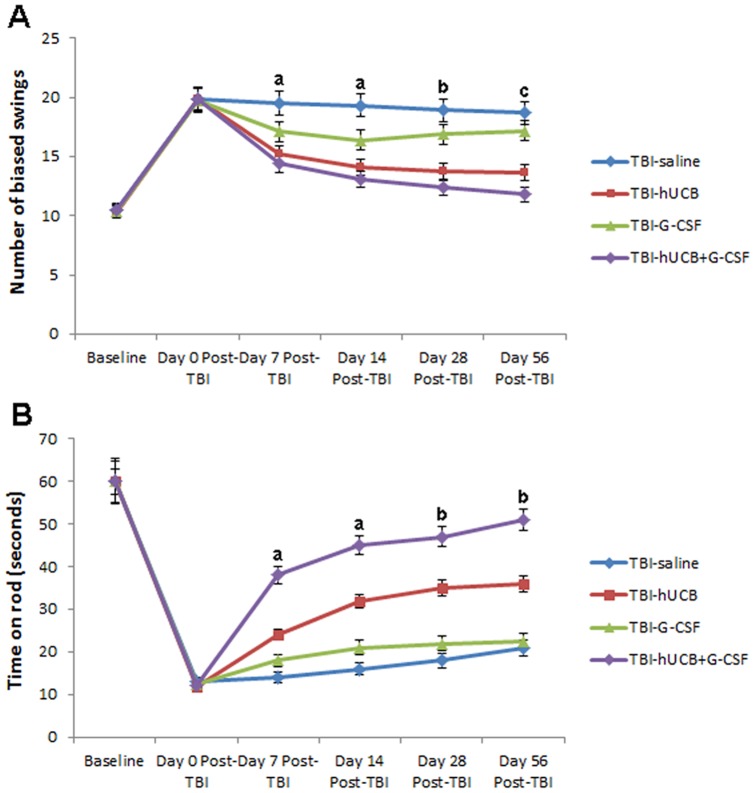
Combined hUCB+G-CSF exert robust functional recovery in chronic TBI. A separate cohort of TBI animals, using the same experimental paradigm above, was subjected to behavioral tests. Elevated body swing tests revealed that animals displayed normal swing activity (average of 10 swings to both left and right side) prior to TBI (Baseline), but exhibited significant swings to one side after TBI (Day 0 post-TBI) (p's<0.05 versus Baseline) (Fig. 4A). At post-TBI day 7 and day 14, TBI-hUCB+G-GSF and TBI-hUCB alone promoted significantly better recovery compared to G-CSF alone, although all three groups performed better than TBI-saline group (a) (p's<0.05). By day 28, TBI-hUCB and TBI-hUCB+G-CSF were the only two groups that displayed significant recovery of normal swing activity (p's<0.05), while TBI-GCSF group reverted to Day 0 post-TBI levels and did not significantly differ from TBI-saline (Fig. 4A) (p>0.05). By day 56, TBI-hUCB+G-GSF and TBI-hUCB alone were still significantly displaying near normal swing activity (p's<0.05 versus TBI-G-CSF or TBI-saline), but the TBI-hUCB+G-GSF showed significantly better recovery than TBI-hUCB alone (c) (p<0.05). In addition TBI-GCSF group did not significantly differ from TBI-saline by day 56 (Fig. 4A) (p>0.05). Rotorod tests revealed that animals learned to balance on the rotating rod (maximum of 60 seconds) prior to TBI (Baseline), but exhibited significant reduction in balancing time after TBI (Day 0) (p's<0.05 versus Baseline) (Fig. 4B). At post-TBI day 7 and day 14, the performance in balancing on the rotating rod across treatment groups showed the following order of best to least recovery: TBI-hUCB+G-GSF>TBI-hUCB alone>G-CSF alone, with all three groups performing better than TBI-saline group (a) (p's<0.05), but the TBI-hUCB+G-CSF displayed the most effective balancing activity at across all time points (p's<0.05 versus all other groups) (Fig. 4B). By day 28 and day 56, only TBI-hUCB and TBI-hUCB+G-CSF were the only two groups that displayed significant recovery of balancing activity (p's<0.05 versus TBI-G-CSF or TBI-saline), while TBI-GCSF group did not significantly differ from TBI-saline (Fig. 4B) (p's>0.05).

ANOVA revealed main effects of treatment (EBST, p<0.001; rotorod, p<0.0001) and time (EBST, p<0.001; rotorod, p<0.0001). ANOVA also revealed treatment by time after TBI interaction effects in both tasks EBST (F_3,27_ = 427.11, p<0.0001) and rotorod test (F_3,27_ = 564.38, p<0.0001). Within-groups comparison across weeks revealed performance in EBST and rotorod test was impaired by moderate TBI immediately after the injury (Day 0, p<0.05), and remained significantly deficient even up to the chronic stage (i.e., 56 days post-TBI, p's<0.05) in TBI-saline treated animals (Fig. 4). In both tasks, while the TBI-saline showed a trend of improved recovery over time, their performance did not reach statistical significance (p's>0.05) compared to Day 0 post-TBI. In contrast, significant recovery in both EBST and rotorod were detected in TBI animals that received monotherapy of hUCB or G-CSF and the combined therapy of hUCB+G-CSF (Fig. 4A, B). In EBST (Figure 4A), hUCB alone and the combined hUCB+G-CSF groups displayed significant improvements across all post-TBI time points (p's<0.05). In contrast, the monotherapy of G-CSF while significantly recovered at days 7–14 post-TBI (p's<0.05), reverted to Day 0 level at day 28 and day 56 post-TBI (p's>0.05). Similarly, TBI-saline treated animals were significantly impaired throughout the post-TBI time points (p's<0.05), whereas TBI animals that received monotherapy of hUCB or G-CSF and the combined therapy of hUCB+G-CSF displayed significant improvement across all post-TBI time points (p's<0.05), and the monotherapy of G-CSF only showed temporary recovery at days 7–14 post-TBI (p's<0.05), then reverting to Day 0 impaired level at day 28 and day 56 post-TBI (p's>0.05). Next, between groups comparisons revealed that the combined therapy of hUCB+G-CSF displayed the most robust recovery in motor performance and showing much better improvement over time compared to the other treatment conditions (p's<0.05) (Fig. 4A). The monotherapy of hUCB was able to mimic the motor performance of the combined hUCB+G-CSF group, but only up to day 28 (p's<0.05), with the combined therapy much more improved than the hUCB monotherapy by day 56 post-TBI (p<0.05). In contrast, the monotherapy of G-CSF while significantly recovered at days 7–14 compared to TBI-saline group, was still displaying impaired motor performance compared to monotherapy of hUCB and combined hUCB+G-CSF group; moreover, the monotherapy of G-CSF was not statistically different from TBI-saline group on days 28 and 56 (p's>0.05) (Fig. 4A). Between groups comparisons in the rotorod tests generally resembled the EBST results, demonstrating the combined therapy promoted the most effective recovery of motor balance and coordination as early as day 7 post-TBI which was maintained, and seemed to display improved recovery over time (throughout the 56-day post-TBI period) compared to all other treatment groups (p's<0.05) (Fig. 4B). The monotherapy of hUCB exhibited significantly better improvement than G-CSF alone and TBI-saline throughout the 56-day post-TBI period (p's<0.05), but not as fully recovered as the combined therapy group at all-time points (p's<0.05) (Fig. 4B). The monotherapy of G-CSF alone showed a short-lived recovery of motor balance and coordination at day 7 and day 14 (p's<0.05), but by day 28 and day 56, this GCSF alone group did not significantly differ from TBI-saline (p's>0.05).

## Discussion

This study demonstrates synergistic therapeutic anti-inflammatory potential of combined therapy of hUCB+G-CSF over monotherapy of hUCB cells or G-CSF in rats exposed to chronic TBI. Chronic TBI is typically associated with major secondary molecular injuries whereby chronic neuroinflammation rampantly instigates an upswing of pro-inflammatory cytokines which further contribute to neuronal cells death, increase reactive oxygen species and downregulate endogenous repair mechanism such as neurogenesis [11], [40]. The involvement of the immune system in the CNS to either stimulate repair or enhance molecular damage has become increasingly recognized as a key component of the pathological onset and progression of many neurological disorders including TBI and neurodegenerative diseases [41]. We and others have shown a marked increase in MHCll+ cells in acute and chronic TBI as well as in many other neuropathological disorders including Alzheimer's disease, multiple sclerosis, Parkinson's disease, and autoimmune diseases [41], [42], [43], [44]. Increased expression of MHCII+ cells is directly correlated with neurodegeneration and cognitive declines of these models [43], [45]. In addition, enhanced positive staining for MHCII+ cells is correlated with the increase of pro-inflammatory cytokines such as TNF-alpha, IL-6, IL-1 and chemokines such as fractalkine. In contrast, decreased MHCII+ cells correlate with reduced aberrant accumulation of reactive oxygen species and an increase of anti-inflammatory cytokines [46], [47]. We are cognizant of the need for cytokine profiling in TBI brain under different treatment conditions. Currently, clinical treatments to specifically target the massive inflammatory secondary insults beyond the original injury, such as chronic white matter injury, are limited and the few that have been utilized have proven to be ineffective when used in long-term exposure of chronic TBI [17], [18], [19].

The present in vivo study sought to examine whether there were any potential synergistic benefits associated with the use of combined therapy of hUCB cells along with factors that would enhance endogenous stem cells such as factor-CSF in a chronic TBI rat model. We show that combined treatment resulted in better reduction of neuroinflammation than monotherapy in the striatum, SVZ and DG, and CC. On the other hand, monotherapy of hUCB cells or G-CSF reduced neuroinflammation in a few gray matter areas; monotherapy had little or no effect in the cortex and thalamus, but worked as well or better in other regions such as the striatum, SVZ and DG, corpus callosum and fornix, showing at best modest amelioration of exacerbated MHC+ cells found in the white matter associated with TBI. Nonetheless, all three treatments of hUCB alone, G-CSF alone, and combined hUCB+G-CSF were able to afford decreased hippocampal cell death, and enhanced neurogenesis in rats exposed to chronic TBI. These observed histological rescue by hUCB alone, G-CSF alone, and combined hUCB+G-CSF translated to behavioral recovery, with the combined therapy affording the most robust improvement in motor performance in treated chronic TBI animals.

These results are in accordance with preclinical data that suggest that intravenous infusion of hUCB cells in ischemic insults such as stroke and TBI was able to block neuroinflammation, improve BBB integrity, as well as decrease brain edema, and increase endogenous angiogenesis [48], [49], [50]. Our results demonstrating a less robust anti-inflammatory effect of G-CSF monotherapy concur with reported preclinical data documenting that the prophylaxis administration of G-CSF alone after brain trauma has small beneficial effects on motor outcomes, on reducing brain edema, decreasing cortical contusion volume or modulating glial cells glutamate concentrations in CSF [51], [52]. However, other studies report that bone marrow mononuclear cells were as neuroprotective as G-CSF alone in an experimental model of spinal cord injury [53]. In addition, it has been shown that either pre-treatment of G-CSF or chronic administration of G-CSF through mini-pump could in fact be highly beneficial at rescuing TBI-associated motor lesions, behavioral impairments, cell death and brain edema [54], [55]. Different G-CSF dosing regimens and disease targets may explain these discrepant results.

In tandem, monotherapy of hUCB cells or G-CSF, and the combined therapy of hUCB+G-CSF cells, significantly reduced the TBI-induced loss of pyramidal neuron cells in the CA3 region of the hippocampus relative to saline alone. Results show that the synergy of the combined therapy is not present since the monotherapy of hUCB cells equally decreased the neuronal cells death in this area of the hippocampus relative to saline alone and monotherapy of G-CSF. Investigations on the neurogenic potential of present treatments revealed that monotherapy of hUCB cells or G-CSF, and the combined therapy of hUCB+G-CSF cells, equally and significantly increased the estimated total number of new neurons in the both neurogenic sites of DG and SVZ relative to saline alone. The influence of hUCB cells or G-CSF injections on neuronal cell loss and neurogenesis has been previously reported; hUCB treatments in models of TBI, aging and stroke studies decrease inflammation and facilitate neurogenesis and angiogenesis [56], [57]. Likewise, G-CSF treatment has been shown to be a potent neurogenic modulator in TBI as well as other neurodegenerative diseases (i.e., hypoxic injury and Alzheimer's disease) in that long-term treatments of G-CSF improve motor function and enhance neurogenesis in the all neurogenic niches [32], [58], [59]. The mechanisms responsible for the beneficial effects of hUCB and G-CSF in the injured brain is not well elucidated, but may likely involve neurogenesis as demonstrated in the present study and other reports [60], [61], [62].

In addition to reducing TBI-mediated histological alterations, the monotherapy of hUCB and G-CSF and their combined therapy led to significant behavioral improvements, indicating the functional benefits of these therapeutics in chronic TBI. That G-CSF alone was only able to produce short-lived improvements in motor function suggests that the present drug regimen (single injection of G-CSF) may need to be optimized, such as repeated treatments especially during the chronic stage to achieve long-lasting benefits. Indeed, studies have shown modest behavioral effects with G-CSF treatments in animal models of neurological disorders [63], [64], [65]. On the other hand, hUCB alone seemed to afford much more improved behavioral outcomes with robust and stable recovery of motor functions in chronic TBI animals, indicating that the stem/progenitor cells may be accomplishing a much more widespread biological action than the drug therapy. Nonetheless, the combination of G-CSF and hUCB resulted in the most effective amelioration of TBI-induced behavioral deficits, suggesting that complementary brain repair processes distinctly or mutually solicited by these two therapies could have mediated the improved behavioral outcome. The mobilization of endogenous stem cells from the peripheral bone marrow by G-CSF [66], [67], coupled by hUCB grafts secretion of growth factors, as well as a potential graft-host integration leading to reconstruction of synaptic circuitry [68], [69], may be multi-pronged regenerative mechanisms triggered by the combined therapy, but not by monotherapy. Additional studies are warranted to elucidate these modes of action associated with combined therapy. Furthermore, the present behavioral tests were limited to motor function, necessitating that future studies should also consider test of cognitive performance which is equally altered by TBI.

A potential mechanism of action by which i.v. injected G-CSF and/or hUCBs influence diverse regions of the brain may be via receptor-mediated transport and paracrine mechanism. G-CSF is a cytokine able to readily mobilize stem cells from bone marrow to the peripheral blood. Previously, it has been shown that these mobilized cells are able to infiltrate injured tissues promoting self-repair of neurons, myocytes and other cells. Evidence suggests that G-CSF can cross the blood brain barrier (BBB) and act upon neurons and glial through G-CSF receptor. Using radioactive labeling, an experiment showed that G-CSF is able to pass through the blood brain barrier of intact animals. The capillaries associated with the BBB express G-CSF receptor and thus the entry of G-CSF could be mediated through this receptor [70]. In our study, gray matter and white matter areas were rescued by the G-CSF and hUCB monotherapies and more synergistically when administered concomitantly. Studies have found that activation of G-CSF receptors on neurons and glial cells downregulates pro-inflammatory cytokines, and increases neurogenesis, among other therapeutic effects (e.g., triggers anti-apoptotic pathways and promotes cerebral angiogenesis), altogether ameliorating sensory and motor deficits in ischemic injuries [71], [72], [73], [74], [75]. In addition, the combination of G-CSF and hUCB cells can promote stemness maintenance, and, under appropriate conditions, guide neural lineage commitment of hUCB in vitro [76], [77]. We and others also recognized the concept of the by-stander effects whereby mobilized bone marrow cells and hUCB cells in the periphery stimulate neuroprotection and brain repair by paracrine mechanism in where cells would secrete trophic factors, growth factors, chemokines and immune-modulatory cytokines to the injured milieu [78], [79], [80], [81], [82], [83]. These studies support our findings on anti-inflammation, enhanced neurogenesis, and increased CA3 cell survival in which monotherapy of G-CSF, hUCB or the combination of both were able to significantly act as neuroprotective agents in our TBI models.

Taken together, these results indicate that while stand-alone therapies of hUCB transplantation and G-CSF treatment demonstrated a moderate degree of efficacy, their combination afforded synergistic robust beneficial effects in neuroinflammation while decreasing neuronal cell death and stimulating endogenous neurogenesis in a chronic model of moderate TBI. The combined therapy also resulted in robust and long-lasting improvements of motor function. In the clinic, chronic TBI has been visualized as worsening histopathology with limited therapeutic manipulation [15], [84]. In the present in vivo study, we demonstrated how well known stand-alone therapies can overcome their own therapeutic limits in chronic stages of TBI when synergy is achieved through combination therapy.
